# Supplementary material for: MetaRibo-Seq measures translation in microbiomes
Source: Nat Commun. 2020 Jun 29;11:3268. doi: 10.1038/s41467-020-17081-z (PMC7324362; doi:10.1038/s41467-020-17081-z)
Supplement: Supplementary file 10 — Supplementary Data 7 [file 41467_2020_17081_MOESM10_ESM.zip › File2/Confidence_High_Taxonomy/156592_out.krona.html]

Javascript must be enabled to view this page.

members
magnitude
magnitudeUnassigned
count
unassigned
taxon
rank

156592\_out

28

2
superkingdom
28

28
976
phylum

28
class
200643

28
171549
order

171552
family
28

genus
1
838

SRS053356\_contig\_number\_contig-100\_5418.5419
28

59823
species
1

SRS104036\_contig\_number\_8061


SRS045195\_contig\_number\_contig-100\_15693.15694
1
species
159272

species
2292054

SRS043841\_contig\_number\_10991
1


SRS013098\_contig\_number\_31877SRS013521\_contig\_number\_12585SRS013687\_contig\_number\_27299SRS014415\_contig\_number\_1351SRS015590\_contig\_number\_8193SRS019285\_contig\_number\_64SRS019910\_contig\_number\_contig-100\_2802.128289SRS023914\_contig\_number\_13361SRS043841\_contig\_number\_11077SRS044535\_contig\_number\_contig-100\_2074.98683SRS047433\_contig\_number\_19398SRS049995\_contig\_number\_33552SRS050752\_contig\_number\_25094SRS065397\_contig\_number\_contig-100\_844.97276SRS077392\_contig\_number\_contig-100\_10157.167132SRS077641\_contig\_number\_17783SRS078176\_contig\_number\_10928SRS103987\_contig\_number\_contig-100\_864.154548SRS1041134\_contig\_number\_contig-100\_1218.68363SRS104327\_contig\_number\_4691SRS143181\_contig\_number\_1647SRS143181\_contig\_number\_2864SRS147614\_contig\_number\_2789SRS148253\_contig\_number\_12825
24
165179
species
